# Epidemiology of ocular trauma in children requiring hospital admission: a 16–year retrospective cohort study

**DOI:** 10.7189/jogh.07.010415

**Published:** 2017-06

**Authors:** Kajo Bućan, Anita Matas, Josipa Marin Lovrić, Darko Batistić, Ivna Pleština Borjan, Livia Puljak, Ivona Bućan

**Affiliations:** 1 Department of Ophthalmology, Split University Hospital Centre, Split, Croatia; 2 Department of Anatomy, Histology and Embryology, School of Medicine, University of Split, Split, Croatia; 3 School of Medicine, University of Split, Split, Croatia

## Abstract

**Background:**

To study the epidemiology of ocular trauma requiring hospital admission in children under 18 years in age.

**Methods:**

This retrospective cohort study included pediatric patients with ocular injuries at the Ophthalmology Department of the Clinical Hospital Centre, Split, Croatia, from 2000 to 2015, classified according to the Birmingham Eye Trauma Terminology.

**Results:**

There were 353 children hospitalized, 82% of boys (mean age 11 years) and 18% of girls (mean age 10 years). The majority of traumas occurred in the outside environment (70%, n = 249), followed by occurrences at home (17%, n = 60), and at a school/nursery (8%, n = 28). Final visual acuity was 6/18 or better in 286 (96%) patients with closed globe injury and in 26 (49%) patients with open globe injury. Severe impairment of vision was found in 12 (4.4%) patients in the closed globe injury group and 26 (49%) patients in the open globe injury group. A statistically significant difference was found between final visual acuity and initial visual acuity in all patients (χ^2^ = 12.8; *P* < 0.001).

**Conclusion:**

The majority of pediatric eye injuries are happening in the outside environment and are preventable. Implementation of well–established safety precautions would greatly reduce this source of visual disability in children.

Ocular trauma is a significant problem throughout the world and, in addition to resultant ocular disability, it also has psychological and social effects on the patient. Approximately 1.6 million people worldwide are blind due to ocular trauma, 2.3 million people have bilateral low vision due to trauma and 19 million have unilateral vision loss [1,2]. Eye trauma constitutes 7% of all bodily injuries and 10–15% of all eye diseases [3].

In the United States, eye trauma is the leading cause of noncongenital unilateral blindness in individuals younger than 20 years of age. The American Academy of Pediatrics (AAP) reported that 66% of all ocular injuries occur in individuals 16 years of age or younger, with the highest frequency occurring between 9 and 11 years of age [4–6]. Most ocular injuries occur in boys, as due to their more aggressive nature, they tend to spend more time playing outdoors and tend to play risky games more frequently than girls. The male–to–female ratio in published studies varies from 3:1 to 5.5:1 [5–7]. Most studies have shown no statistically significant difference between affected eyes [8].

Various studies have reported that children account for 12.5–33.7% of all admissions for eye injury. Trauma is clearly one of the most important preventable causes of childhood blindness [9]. The frequency of hospitalization due to ocular trauma differs between developed and developing countries; for example, the rate is 8 per 100 000 people in Scotland and 33 per 100 000 in Guiana [10].

The standardized classification of eye trauma is useful for ophthalmologists and provides the means for simple and enhanced communication regarding particular patient features [11].

Kuhn et al. [12] developed a prognostic model, the ocular trauma score (OTS), to predict the visual outcome of patients in all age groups after open globe and closed globe ocular injuries. They analyzed more than 2500 eye injuries from the United States Eye Injury Registry and the Hungarian Eye Injury Registry and evaluated more than 100 variables with the goal of identifying specific predictors. In the calculation of OTS, a numerical value is assigned to the following six variables: initial visual acuity (VA), globe rupture, endophthalmitis, perforating injury, retinal detachment, and relative afferent pupil defect (RAPD). The scores are then divided into five categories that provide the probabilities of attaining a range of VAs after injury.

Numerous studies have evaluated various aspects of ocular trauma. The purpose of this study was to analyze epidemiology of all eye injuries in children who required admission to the Ophthalmology Department of the University of Split Hospital Centre, Croatia, from 2000 to 2015.

## METHODS

Medical records of all patients aged 18 years or younger who sustained serious eye injuries requiring admission to the Ophthalmology Department at University of Split Hospital Centre between 2000 and 2015 were reviewed. Ethics committee of University of Split Hospital Centre, Split, Croatia, approved the study to be reported. All study procedures adhered to the recommendations of the Declaration of Helsinki.

University of Split Hospital Centre is the only referral hospital for the population of the Split–Dalmatia County (south Croatia). The population of the province as determined in the 2011 census was 455 242. The number of children in the province was 107 316, which consisted of 54 768 boys and 52 548 girls. The distributions of age, gender and socioeconomic status of children in this county were comparable to those of the entire Croatian population.

The study included 353 children treated acutely in the hospital. The following data were recorded for each patient: age, sex, date of injury, site of incident, cause of injury (accidental self–inflicted injury vs injury by another person), visual acuity, diagnosis, associated injuries and treatment.

The injuries were classified according to Birmingham Eye Trauma Terminology (BETT) [13] as the following: closed eye globe injuries and open eye globe injuries (penetration, perforation, intraocular foreign body injury and rupture).

The data were collected, entered and processed using the statistical package SPSS version 15 (SPSS Inc., Chicago, IL, USA). The results were interpreted using a significance level of *P* < 0.05. The χ^2^ test, McNemar test, Kruskal-Wallis test and Mann-Whitney U test were used in the analysis.

## RESULTS

**Figure 1:**
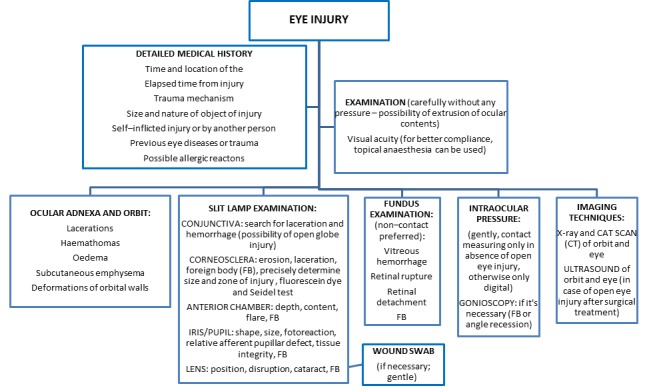
Standard operative procedures for managing eye injuries at the Department of Ophthalmology, University of Split Hospital Centre (Ivna Pleština Borjan).

**Figure 2:**
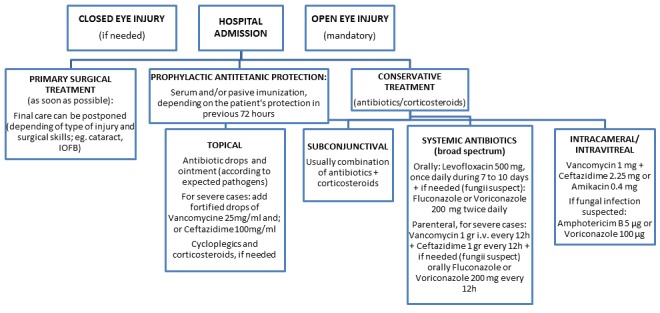
Standard operative procedures for hospital admission of eye injuries at the Department of Ophthalmology, University of Split Hospital Centre (Ivna Pleština Borjan).

A total of 353 children with eye injuries were admitted to the clinic during the 16–year study period; there were 290 (82%) boys and 63 (18%) girls, yielding a male–to–female ratio of 5:1. Assessing and treatment of eye injuries and indications of hospital admission by algorithms that we developed for managing of eye trauma in our Department are presented in **Figure 1** and **Figure 2**. The mean age at admission was 11 years among boys and 10 years among girls. The right eye was involved in 174 (49%) cases, and the left eye was involved in 177 (50%) cases. Binocular injury was found in 1 child (0.2%). There was no statistically significant difference between injuries of the right eye and the left eye according to age (χ^2^ = 2.33; *P* = 0.506). In average duration of hospitalization was 9.8 days with median of 5 to 13 days (**Table 1**).

**Table 1:** Eye injuries in children according to the type of injuries and duration of hospitalization

Type of injury	No of children (%)	Duration of hospital admission in days – median (min–max)
**Closed globe:**		
Contusion	279 (79)	7.2 (5–8)
Lamellar laceration	20 (5.7)	9.3 (8–11)
**Open globe:**		
Penetration	48 (13.6)	9.1 (6–12)
IOFB*	2 (0.57)	10.9 (9–13)
Perforation	2 (0.57)	11.1 (10–13)
Rupture	2 (0.57)	11.1 (10–13)

IOFB – intraocular foreign body

The injured children were divided into the following four age groups: 0–4 years (infants and preschool), 5–9 years of age, 10–14 years (elementary school), and 15–18 years (high school). The largest number of injuries (39%) occurred among children aged 10–14 years, followed by those aged 5–9 years (34%), and those aged 15–18 years (19%); the fewest injuries occurred among children aged 0–4 years (8.5%) (**Table 2**).

**Table 2:** Age and gender of children with eye injuries

Age (years)	Total (n = 53) (%)	Male (n = 290) (82%)	Female (n = 63) (18%)
0–4	30 (8.5)	27 (9.3)	3 (4.8)
5–9	119 (33.7)	97 (33.5)	22 (34.9)
10–14	137 (38.8)	105 (36.2)	32 (50.8)
15–18	67 (19)	61 (21)	6 (9.5)

The cumulative incidence of eye injuries among boys was 530/100 000, and among girls 120/100 000 (**Table 3**). The cumulative incidence of eye injuries among boys was 4.4 times higher than that among girls.

**Table 3:** Cumulative incidence of children with eye injuries/100 000, from 2000 to 2015

Age (years)	Total (95% CI)	Boys (95% CI)	Girls (95% CI)	*P*–value*
0–4	120 (80–170)	220 (150–320)	30 (10–80)	<0.001
5–9	490 (410–590)	780 (640–950)	190 (130–290)	<0.001
10–14	480 (410–570)	720 (590–870)	230 (160–320)	<0.001
15–18	220 (170–280)	400 (310–510)	40 (20–90)	<0.001

CI – confidence interval

*χ^2^ test.

Compared to girls, the cumulative incidence of eye injuries among boys was 7 times higher among children age 0–4 years (χ^2^ = 16.3, *P* < 0.001), 4 times higher among children age 5–9 years (χ^2^ = 42, *P* < 0.001), 3 times higher among children age 10–14 years (χ^2^ = 36, *P* < 0.001) and 10 times among children age 15–18 years (χ^2^ = 41, *P* < 0.001). In all age groups, boys had higher incidence of eye injuries compared to girls.

The majority of injuries occurred during spring and summer (**Table 4**). Compared to autumn, there were 1.6 times more eye injuries during spring and 1.8 times more eye injuries during summer (χ^2^ = 13.6; *P* = 0.035).

**Table 4:** Number of children (%) with eye injuries according to season

Season	Total (%) (n = 353)	*P*–value*
Winter	86 (24.4)	0.035
Spring	100 (28.3)	0.035
Summer	106 (30.0)	
Autumn	61 (17.0)	

*χ^2^ test.

The majority of traumas occurred in the outside environment (outside of the home, school or nursery) (70%, n = 249), followed by at home (17%, n = 60), at school/nursery (8%, n = 28), at a sporting area (4%, n = 14) and in traffic (1%, n = 2) (**Table 5**).

**Table 5:** Number of children and age – median according to site of injury

Site of injury	Total *P*–value* n (%)	Age – median *P*–value† (min–max)
Home	60 (17)‡	8 (2–17)‡
School/nursery	28 (8)	12 (3–17)
Outside home/school/nursery	249 (70)	11 (2–18)
Traffic	2 (1)	
Sport	14 (4)	12 (10–18)

*χ^2^ test.

†Kruskal–Wallis test.

‡*P* < 0.001.

Children were 9 times more likely to be injured in the outside environment compared to school and nursery, and they were 4 times more likely to be injured in the outside environment than in the home (χ^2^ = 412; *P* < 0.001). Our study showed a statistically significant difference in the age of children according to the site of injury (χ^2^ = 25.1; *P* < 0.001); the median age of children injured at home was 4 years lower than that of children injured in school (Z = 3.15, *P* = 0.02), 3 years lower than that of children injured outside the home (Z = 4.4, *P* < 0.001), and 4 years lower than that of children who were injured during sports (Z = 3.8, *P* < 0.001) (**Table 5**).

There were 112 (32%) children with accidental self–inflicted injury, and their median age was 10 years (range: 2–18), while 239 (68%) children who were injured by another person, and their median age was 11 years (range: 2–18).

Significant difference between the age of children with accidental self–inflicted injury and those injured by another person was not observed (χ^2^ = 1.02; *P* = 0.307) (**Table 6**).

**Table 6:** Number of children (%) with eye injury according to age in relation to person who caused an injury

Age (years)	Total n (%)	Self–inflicted injury n (%)	Injured by other person n (%)		*P*–value*
0–4	30 (8.5)	13 (12)	17 (7)	0.307	
5–9	119 (33.7)	39 (35)	79 (33)		
10–14	137 (38.8)	37 (33)	100 (42)		
15–18	67 (19)	23 (20)	44 (18)		

*χ^2^ test.

With regard to the type of injury, there were 299 (85%) closed eye injuries and 54 (15%) open eye injuries. The median age of children with closed injuries was 2 years higher than the median age of children with open eye injuries (Z = 2.98, *P* = 0.03) (**Table 7**).

**Table 7:** Age and types of injuries in children with eye injuries

Type of injury	No of children (%)		Age – median (min–max)		*P*–value*	
Closed globe:		11 (2–18)		0.03	
Contusion	279 (79)				
Lamellar laceration	20 (5.7)				
Open globe:		9 (2–17)			
Penetration	48 (13.6)				
IOFB	2 (0.57)				
Perforation	2 (0.57)				
Rupture	2 (0.57)				

IOFB – intraocular foreign body

*Mann–Whitney U test.

Initial visual acuity was normal or mildly impaired (better than 0.3) in 208 (70%) patients with closed globe and 13 (25%) patients with open globe injury (**Table 8**).

**Table 8:** Number of children (%) with eye injury according to visual acuity in relation to the type of eye injury (closed globe–open globe) at hospitalization

	**Type of injury**		
**Initial visual acuity**	**Closed globe**	**Open globe**	**Total**
Normal (0.9–1.0)	86 (28.9)	4 (7.6)	90
Mildly impaired (0.3–0.8)	122 (41)	9 (17)	131
Poor (0.02–0.25)			
Moderate (0.125–0.25)	24 (8)	4 (7.6)	28
Serious (0.05–0.1)	19 (6.4)	7 (13.3)	26
Deep (0.02–0.04)	26 (8.7)	8 (15.1)	34
Semi–blindness (light perception to 0.01)	20 (38)	20 (38)	40
Blindness (no light perception)	1 (0.3)	0	1
Total	n = 299 (85)	n = 54 (15)	n = 353

Final visual acuity was greater than or equal to 0.3 in 286 (96%) patients with closed globe injuries and 26 (49%) patients with open globe injuries. Severe vision impairment (worse than 0.3) was found in 12 (4.4%) patients with closed globe injuries and 26 (49%) patients with open globe injuries (**Table 9**).

**Table 9:** Number of children (%) with eye injury according to final visual acuity in relation to initial visual acuity at release from hospital

	**Type of injury**		
**Final visual acuity**	**Closed globe**	**Open globe**	**Total**
Normal (0.9–1.0)	239 (80)	9 (17)	248
Mildly impaired (0.3–0.8)	47 (16)	17 (32)	64
Poor (0.02–0.25)			
Moderate (0.125–0.25)	2 (0.7)	6 (11.3)	8
Serious (0.05–0.1)	6 (2)	7 (13.2)	13
Deep (0.02–0.04)	2 (1)	5 (9.4)	7
Semi–blindness (light perception to 0.01)	1 (0.33)	8 (15.1)	9
Blindness (no light perception)	1 (0.33)	0	1
Total	n = 299 (85)	n = 54 (15)	n = 353

Overall improvement of visual acuity of all patients at the end of the treatment was significantly higher compared to initial visual acuity (χ^2^ = 12.8; *P* < 0.001). Compared to initial visual acuity, visual acuity improved in 86% of patients and remained the same in 14% of patients; no patient experienced deteriorated visual acuity (**Table 10**).

**Table 10:** Number of children according to visual acuity after the treatment in relation to initial visual acuity

	Visual acuity – initial					
**Visual acuity – final**	Normal	Mildly impaired	Poor moderate	Poor serious	Poor deep	Semi–blindness
	n = 90	n = 131	n = 28	n = 26	n = 34	n = 40
Normal	90	108	14	15	14	7
Mildly impaired		23	12	5	13	10
Poor moderate			2	2	3	1
Poor serious				4	1	8
Poor deep					3	5
Semi–blindness						9
Improvement; n (%)		108 (92)	26 (93)	22 (85)	31 (91)	31 (77)

## DISCUSSION

Ocular injuries are the most common cause of acquired uniocular blindness in children. Pediatric ocular injuries differ from those of adults in many ways. Ocular trauma in children is mainly accidental and has an age–specific pattern [14].

In this study, pediatric ocular trauma occurred 4.5 times more often in boys than in girls. Boys are usually more susceptible to ocular damage due to the nature of their activities and presumed less supervision by their families, similar to results from other studies [1,4,6,10,14]. In our study, the highest incidence of eye injuries occurred among children age 10 to 14 years, which is also similar to studies from other settings [1,4,15–17].

In contrast to the above findings, Al–Bdour and Azab reported the highest incidence of injuries among children aged 6 to 10 years. Children in this age group are relatively immature and exposed to varying surroundings that make them more vulnerable to injuries [6,14].

Both eyes were affected equally. Bilateral ocular injuries were observed only in 1 patient. This is in accordance with most other studies, where ocular trauma plays a minor role in bilateral blindness compared to its major role in unilateral blindness [6,14].

The majority of injuries occurred during spring and summer, which is similar to results reported elsewhere [18]. The summer vacation months accounted for a disproportionate number of eye injuries received throughout the year. The summer offers children the opportunity to spend more time outside and to have more freedom to play with potentially dangerous objects. Furthermore, the lack of school during the summer months may adversely affect the time children are supervised by adults.

The present study showed that ocular injury occurred most commonly in the outside environment, with the home as a second most common site; this is consistent with observations similar to results reported elsewhere [16]. It speaks in favor of possible lack of adult supervision while children play outside. A study conducted by Aghadoost et al. showed that most injuries happened at home [10]. A study in North Jordan showed that eye injuries occurring during sports and play were the most common [6]. In Canada, eye injuries occurred at a number of locations, with the majority occurring at homes, followed by schools and other residences [18].

Our study showed a statistically significant difference between the age of children according to the site of injury. Children injured at home were approximately 4 years younger than children injured at school and during sports and approximately 3 years younger than those injured outside the home. These results were expected because younger children spend more time at home.

In our study, more than two–thirds of patients were injured by another person, and this did not differ by age group. In other studies, most eye injuries were reported as being unintentional, though there were instances in which the injury happened during a physical altercation [18].

With respect to the BETT classification in our study, closed globe injury occurred five and a half times more frequently than open globe injury, and this is similar to results reported elsewhere [19]. We showed that children with open eye injuries were 2 years younger than children with closed eye injuries. The average age of children was 11 years among those with closed globe injuries and 9 years among those with open globe injuries. We considered that young children are more prone to open globe injuries due to their natural desire to explore, their lack of fear of danger and their limited ability to avoid danger. Jandeck et al. showed that the average age of children with open globe injuries was 8.7 years, which is similar to our results [20]. The standardized classification of eye trauma is useful for ophthalmologists and provides the means for simple and enhanced communication about particular patient features [11].

Initial visual acuity was normal or mildly impaired in 70% of patients with closed globe injuries and 25% of patients with open globe injuries. Decreased visual acuity occurred more in open globe injuries and with ruptured globes. Final visual acuity was normal or mildly impaired in 96% of patients with closed globe injury group and 49% of patients with open globe injuries. Severe vision impairment was found in 4.4% of patients with closed globe injuries and 49% of patients with open globe injuries. Among those with open globe injuries, penetrating injuries were the most common. Penetrating injuries, in general, carry a poorer prognosis, and they are more likely to require surgery and result in long–term visual impairment.

We found a statistically significant difference between final and initial visual acuity. Good visual acuity at presentation and early primary repair were important factors for better final visual outcome. Compared to blunt injuries, penetrating injuries generally resulted in poorer visual outcomes. Posterior segment involvement adversely affects visual results [14,20,21].

Although our study covers a relatively long time period of 16 years, the retrospective nature is an acknowledged weakness of this study.

In conclusion, severe ocular trauma in children that requires hospitalization is mainly accidental and has an age–specific pattern. In general, children are more susceptible to eye injuries due to their immature motor skills, limited common sense and natural curiosity. A safe environment should be maintained for children. The majority of eye injuries in children are preventable, which reflects the importance of health education, adult supervision and application of appropriate measures to reduce the incidence and severity of trauma.
